# Correlation between body composition and white matter hyperintensity in patients with acute ischemic stroke

**DOI:** 10.1097/MD.0000000000036497

**Published:** 2023-12-15

**Authors:** Bin Wu, Dong Huang, Ziwei Yi, Fang Yu, Li Liu, Xianbi Tang, Kaiquan Jing, Jiangli Fan, Chuzheng Pan

**Affiliations:** a Department of Neurology, Hunan University of Medicine General Hospital, Huaihua, People’s Republic of China; b The Advanced Stroke Center of China, Huaihua, People’s Republic of China; c Jishou University, Jishou, People’s Republic of China; d The Forth People’s Hospital of Huaihua, Huaihua, People’s Republic of China.

**Keywords:** erector spinae muscle, subcutaneous adipose tissue, bone density, white matter hyperintensity, acute ischemic stroke

## Abstract

White matter hyperintensity (WMH) burden is associated with a higher risk of ischemic stroke. The relationship between WMH and obesity is somewhat controversial which might be interfered by different body composition such as skeletal muscle, fat and bone density. However, few researchers have evaluated the relationship between WMH burden and disaggregated body constituents in acute ischemic stroke (AIS) patients systematically. A total of 352 AIS patients were enrolled in this study. The subcutaneous adipose tissue, erector spinae muscle area and bone density were evaluated on the computed tomography scanning. The burden of WMH was evaluated using the Fazekas scale based on the fluid-attenuated inversion recovery sequence. The severity of overall WMH was defined as none-mild WMH (total Fazekas score 0–2) or moderate-severe WMH (total Fazekas score 3–6). Based on the severity of periventricular WMH (P-WMH) and deep WMH, patients were categorized into either a none-mild (Fazekas score 0–1) group or a moderate-severe (Fazekas score 2–3) group. We found that patients with moderate-severe WMH showed lower bone density and smaller erector spinae muscle area and subcutaneous adipose tissue than none-mild. The logistic regression analysis showed that the bone density was independently associated with moderate-severe overall WMH (odds radio = 0.98, 95% confidence interval, 0.972–0.992, *P* < .001) and similar results were found in the analyses according to P-WMH (odds radio = 0.98, 95% confidence interval, 0.972–0.992, *P* < .001). These findings suggest that among the AIS body composition, the bone density is independently associated with the severity of overall WMH and P-WMH.

## 1. Introduction

Stroke is the fifth leading cause of mortality and a major cause of morbidity in the world.^[1]^ White matter hyperintensity (WMH) is the most common radiological marker of small vessel disease,^[2]^ and mounting evidence has shown that WMH burden is related to the risk of stroke, recurrent stroke, and poorer outcomes after stroke.^[3,4]^ Therefore, identifying risk factors is crucial to improve our understanding of the etiology and consequences of WMH in patients with ischemic stroke.

WMH is a subclinical pathology, which represents tissue rarefaction or myelin pallor arising from a loss of axon or gliosis.^[5]^ Many studies have suggested several potential mechanisms of the development of WMH (e.g. diffuse hypoperfusion, endothelial dysfunction, glymphatic blockage), however, this process is still not understood.^[6,7]^ Obesity is defined as abnormal or excessive fat accumulation and is associated with atherosclerosis, subclinical inflammation, and ischemic stroke.^[8]^ Recently, osteosarcopenic obesity syndrome has been identified as a deleterious condition including loss of bone, loss of muscle mass and increased body fat (even if they are not obese by conventional measures).^[9]^ WMH, a precursor to dementia and stroke, could have a close association with obesity, but the association of body mass index (BMI) with WMH is somewhat controversial.^[10,11]^ This phenomenon might result from different body composition of each patient.

In this study, we investigated the relationship between the different body composition measured by computed tomography (CT) and WMH in acute ischemic stroke (AIS) patients.

## 2. Materials and methods

### 2.1. Study participants

This study included consecutive patients with AIS between January 2022 and October 2022. We recruited 352 patients with AIS confirmed by magnetic resonance imaging (MRI) of the brain within 14 days of symptom onset. The other inclusion criterion was age ≥ 18 years. We excluded patients with disabilities (Modified Rankin Scale score ≥ 2) before stroke onset and those without fluid-attenuated inversion recovery sequence (FLAIR). This study was approved by the ethics committee of the Hunan University of Medicine General Hospital.

### 2.2. Clinical assessments

We assessed demographic characteristics and medical history, including age, sex, BMI, vascular risk factors (i.e., hypertension, diabetes mellitus, hyperlipidemia, coronary heart disease, history of stroke, smoking and moderate or heavy drinking). Kidney function, fasting blood glucose, homocysteine, HbAlc and serum lipids were determined from each patient after admission to hospital. The use of hypotensive and hypoglycemic drugs were also included. Hyperlipidemia was defined as the use of lipid lowering agents, or ≥ 5.7 mmol/L in total cholesterol levels, or ≥ 1.7 mmol/L triglyceride levels. The National Institutes of Health Stroke Scale was used to assess the severity of stroke.^[12]^ Laboratory examinations regarding the glucose profile, lipid profile and HbAlc were performed after 12 hours of overnight fasting.

### 2.3. The measurements of skeletal muscle, fat and bone density

**Figure 1.**
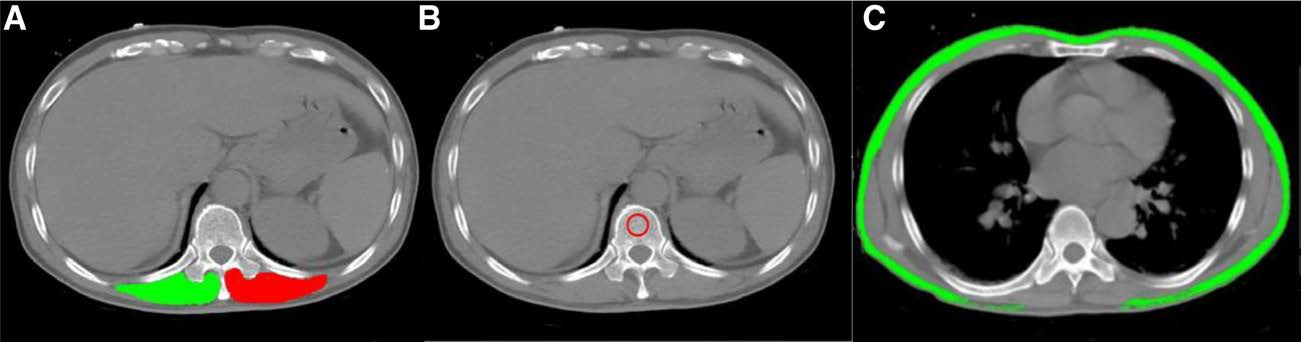
Sample CT scans used to determine muscle area in our cohort. (A) ESMA measured at the T12 level: green indicate right muscle and red, left muscle. (B) Bone density measured in the region of interest (ROI) indicated with red circle, at the T12 level. (C) SAT measured at the T7-8 level, highlighted in green. CT = computed tomography.

Erector spinae muscle area (ESMA) and bone density measurement was performed using a single axial slice of the CT scan with an in-house software. The analysis of ESMA was performed on a single axial chest CT image at the level of the lower margin of the 12th thoracic vertebrae^[13]^ and the cross-sectional area was presented as the sum of the right and left muscles expressed in square centimeters (Fig. 1A). Bone density was measured at the T12 level by determining the Hounsfield attenuation units value^[14]^ (Fig. 1B). Adipose tissue parameters were quantified using SliceOmatic (V5.0, Tomovision, Magog, Canada) software. We derived cross-sectional CT images of the T7-8 vertebral level from the PACS system in the radiology department and studied them in DICOM format. Tissue segmentation was performed using Hounsfield unit thresholds of −190 to −30 for subcutaneous adipose tissue (SAT)^[14,15]^ (Fig. 1C). These tomographic levels were selected as they simultaneously capture the 3 body constituents of interest, which can be analyzed with described methods and accessed with either chest or abdomen CTs.

### 2.4. FLAIR MRI assessment of WMH

Periventricular WMH (P-WMH) and deep WMH (D-WMH) were assessed on FLAIR images using the Fazekas scale, which ranges from 0 to 3. We categorized the severity of P-WMH and D-WMH as none-mild (Fazekas score 0–1) or moderate-severe (Fazekas score 2–3).^[16]^ The total Fazekas score was classified based on the sum of P-WMH and D-WMH (range 0–6). The severity of overall WMH was identified as follows: none-mild WMH (Fazekas score 0–2) or moderate-severe WMH (Fazekas score 3–6).^[17]^ The assessments of ESMA, bone density and SAT were reviewed by 2 independent radiologists who were blinded to the clinical history, and the final decision was made through a consensus meeting after discussion.

### 2.5. Statistical analysis

Data analyses were performed by SPSS 23.0 for Windows (IBM, SPSS). The participants were dichotomized according to WMH burden into none-mild and moderate-severe groups using the Fazekas scores. Data were expressed as mean ± SD and n (%) for continuous and categorical variables, respectively. Categorical variables were compared using the 2-tailed Pearson χ^2^ and non-categorical variables were compared using Student *t* test or Mann–Whitney *U* test. Variables with a univariate *P* values < .10 were selected for further multivariate analysis. All *P* values were defined as significant at *P* < .05. Analyzed variables from logistic regression tests are presented as odds ratios (OR) with 95% confidence intervals (CI).

## 3. Results

### 3.1. Clinical characteristics of patients with AIS

A total of 352 patients (256 men and 96 women aged 65.4 ± 11.0 years) with AIS were enrolled in our study. The clinical and demographic characteristics of these patients were shown in Table 1. Hypertension was recognized in 69.3% of patients, smoking in 30.7%, diabetes mellitus in 34.1% and hyperlipidemia in 38.1%.

**Table 1:** Baseline characteristics among patients with acute ischemic stroke.

Characteristics	Values
Age (yr), mean ± SD	65.4 ± 11.0
Male gender (%)	256 (72.7)
BMI, kg/m^2^	24.1 ± 3.0
Hypertension (%)	244 (69.3)
Hyperlipidemia (%)	134 (38.1)
Diabetes mellitus (%)	120 (34.1)
History of CAD (%)	44 (12.5)
History of stroke (%)	66 (18.8)
AF (%)	32 (9.1)
Smoking (%)	108 (30.7)
Moderate or heavy drinking (%)	48 (13.6)
NIHSS	3.4 ± 3.8
Total cholesterol (mmol/L)	4.5 ± 2.9
LDL cholesterol (mmol/L)	2.5 ± 0.9
HDL cholesterol (mmol/L)	1.1 ± 0.3
Triglyceride (mmol/L)	1.8 ± 1.5
BUN (mmol/L)	6.2 ± 2.5
Uric acid (µmol/L)	333.1 ± 100.5
Creatinine (µmol/L)	85.5 ± 58.0
Fasting blood-glucose (mmol/L)	7.0 ± 3.4
HbA1c (%)	6.9 ± 3.6
Homocysteine (µmol/L)	15.5 ± 8.5
Fazekas score	3.2 ± 1.4
Bone density (HU)	133.2 ± 41.3
ESMA (cm^2^)	30.1 ± 8.8
SAT (cm^2^)	104.7 ± 48.3
SAT/ESMA	3.9 ± 2.3

AF = atrial fibrillation, BMI = body mass index, BUN = blood urea nitrogen, CAD = coronary artery disease, ESMA = erector spinae muscle area, HDL = high-density lipoprotein, HU = Hounsfield attenuation units, LDL = low-density lipoprotein, NIHSS = the National Institutes of Health Stroke Scale, SAT = subcutaneous adipose tissue.

### 3.2. The association between body composition and the severity of overall WHM

There were 115 (32.7%) patients with none-mild overall WMH (total Fazekas score 0–2) and 237 (67.3%) patients with moderate-severe overall WMH (total Fazekas score 3–6). When compared with patients with none-mild WMH, patients with moderate-severe WMH were older (*P* < .001) and had a higher frequency of hypertension (*P* < .001), history of stroke (*P* = .002), hypotensive drugs (*P* < .001), and higher levels of creatinine (*P* = .040). Lower levels of total cholesterol (*P* = .028), low-density lipoprotein cholesterol (*P* = .019), bone density (*P* < .001), ESMA (*P* < .001) and FAT/ESMA (*P* = .006) were observed in moderate-severe WMH subjects (Table 2). From the logistic regression model, history of stroke (OR = 2.36, 95% CI, 1.076–5.188, *P* = .032), creatinine (OR = 1.02, 95% CI, 1.003–1.032, *P* = .014) and bone density (OR = 0.98, 95% CI, 0.971–0.990, *P* < .001) were proven to be the factors that were independently associate with moderate-severe overall WMH (Table 3).

**Table 2:** Baseline characteristics of all patients according to the degree of overall WMH.

	None-mild WMH (n = 115)	Moderate-severe WMH (n = 237)	*P*
Age (yr), mean ± SD	60.6 ± 12.2	67.7 ± 9.5	.000
Male gender (%)	88 (76.5%)	168 (70.9%)	.266
BMI, kg/m^2^	24.2 ± 3.0	24.1 ± 3.0	.804
Hypertension (%)	61 (53.0%)	183 (77.2%)	.000
Hyperlipidemia (%)	48 (41.7%)	85 (35.9%)	.286
Diabetes mellitus (%)	34 (29.6%)	86 (36.3%)	.212
History of CAD (%)	9 (7.8%)	35 (14.8%)	.065
History of stroke (%)	11 (9.6%)	55 (23.2%)	.002
AF (%)	13 (11.3%)	19 (8.0%)	.314
Smoking (%)	31 (27.0%)	77 (32.5%)	.291
Moderate or heavy drinking (%)	13 (11.3%)	35 (14.8%)	.374
Hypotensive drugs	41 (35.7%)	133 (56.1%)	.000
Hypoglycemic drugs	30 (26.1%)	72 (30.4%)	.405
NIHSS	3.4 ± 3.5	3.4 ± 3.9	.931
Total cholesterol (mmol/L)	5.0 ± 4.9	4.2 ± 1.0	.028
LDL cholesterol (mmol/L)	2.6 ± 0.8	2.4 ± 0.9	.019
HDL cholesterol (mmol/L)	1.0 ± 0.3	1.1 ± 0.3	.312
Triglyceride (mmol/L)	1.9 ± 1.6	1.7 ± 1.4	.450
BUN (mmol/L)	5.8 ± 2.0	6.3 ± 2.7	.087
Uric acid (µmol/L)	325.1 ± 102.3	337.0 ± 99.6	.299
Creatinine (µmol/L)	76.4 ± 20.7	90.0 ± 68.8	.040
Fasting blood-glucose (mmol/L)	6.9 ± 3.3	7.0 ± 3.4	.691
HbA1c (%)	7.2 ± 5.7	6.8 ± 2.0	.389
Homocysteine (µmol/L)	14.4 ± 7.6	16.0 ± 8.9	.126
Bone density (per 100 HU)	155.2 ± 40.6	122.5 ± 37.2	.000
ESMA (cm^2^)	33.4 ± 10.0	28.4 ± 7.6	.000
Fat (cm^2^)	100.6 ± 45.4	106.7 ± 49.6	.274
FAT/ESMA	3.4 ± 2.1	4.1 ± 2.4	.006

AF = atrial fibrillation, BMI = body mass index, BUN = blood urea nitrogen, CAD = coronary artery disease, ESMA = erector spinae muscle area, HDL = high-density lipoprotein, HU = Hounsfield attenuation units, LDL = low-density lipoprotein, NIHSS = the National Institutes of Health Stroke Scale, SAT = subcutaneous adipose tissue.

**Table 3:** Logistic regression analyses of the association between different body composition and overall WMH.

	WMH			
	*P* value	OR	95% CI for OR	
			Lower	Upper
Age	.924	1.00	0.967	1.038
Hypertension (%)	.051	1.99	0.998	3.977
History of CAD (%)	.296	1.58	0.671	3.713
History of stroke (%)	.032	2.36	1.076	5.188
Hypotensive drugs	.116	1.71	0.876	3.317
Total cholesterol (mmol/L)	.470	0.84	0.519	1.353
LDL cholesterol (mmol/L)	.997	1.00	0.573	1.742
BUN (mmol/L)	.192	0.91	0.780	1.051
Creatinine (mmol/L)	.014	1.02	1.003	1.032
Bone density (HU)	.000	0.98	0.971	0.990
ESMA (cm^2^)	.064	0.96	0.917	1.003
SAT/ESMA	.823	1.02	0.867	1.196

BUN = blood urea nitrogen, CAD = coronary artery disease, CI = confidence interval, ESMA = erector spinae muscle area, HU = Hounsfield attenuation units, LDL = low-density lipoprotein, OR = odds ratios, SAT = subcutaneous adipose tissue.

### 3.3. The association between body composition and the severity of WHM according to the location of WMH

To further explore the relationship between body composition and different area of WHM burden, we divided all patients into a P-WMH group and a D-WMH group. We categorized the severity of P-WMH and D-WMH as none-mild (Fazekas score 0–1) and moderate-severe (Fazekas score 2–3), respectively. There were 100 (28.4%) patients with none-mild P-WMH and 252 (71.6%) patients with moderate-severe P-WMH. Compared with patients with none-mild P-WMH, patients with moderate-severe P-WMH were older (*P* < .001) and had a higher frequency of hypertension (*P* < .001), diabetes mellitus (*P* = .023), history of coronary heart disease (*P* = .049), history of stroke (*P* = .001), hypotensive drugs (*P* < .001) and hypoglycemic drugs (*P* = .009) and lower levels of total cholesterol (*P* = .009), low-density lipoprotein cholesterol (*P* = .005), bone density (*P* < .001), ESMA (*P* < .001) and SAT/ESMA (*P* < .001) (Supplementary Table 1, http://links.lww.com/MD/L22). When classified by D-WMH, 242 (68.8%) and 110 (31.2%) patients were in the none-mild and moderate-severe D-WHM groups, respectively. Patients with moderate-severe D-WMH were older (*P* < .001) and had low levels of bone density (*P* < .001), ESMA (*P* < .001) and men (*P* = .039) (Supplementary Table 2, http://links.lww.com/MD/L23). The history of stroke (OR = 2.60, 95% CI, 1.058–6.381, *P* = .037) and bone density (OR = 0.98, 95% CI, 0.972–0.992, *P* < .001) were independently associated with severe P-WMH (Table 4). Age (OR = 1.04, 95% CI, 1.006–1.073, *P* = .020) were independently associated with severe D-WMH (Table 5).

**Table 4:** Logistic regression analyses of the association between different body composition and P-WMH.

	P-WMH			
	*P* value	OR	95% CI for OR	
			Lower	Upper
Age	.638	1.01	0.972	1.048
Hypertension (%)	.082	1.92	0.921	4.006
Hyperlipidemia (%)	.943	0.98	0.513	1.858
Diabetes mellitus (%)	.570	0.74	0.265	2.077
History of CAD (%)	.313	1.63	0.633	4.177
History of stroke (%)	.037	2.60	1.058	6.381
Hypotensive drugs	.064	1.99	0.960	4.124
Hypoglycemic drugs	.110	2.46	0.817	7.400
Total cholesterol (mmol/L)	.157	0.63	0.330	1.196
LDL cholesterol (mmol/L)	.691	1.17	0.543	2.514
Creatinine (mmol/L)	.069	1.01	0.999	1.026
Bone density (HU)	.000	0.98	0.972	0.992
ESMA (cm^2^)	.119	0.95	0.881	1.015
SAT (cm^2^)	.532	1.01	0.986	1.029
SAT/ESMA	.818	0.94	0.537	1.633

BUN = blood urea nitrogen, CAD = coronary artery disease, CI = confidence interval, ESMA = erector spinae muscle area, HU = Hounsfield attenuation units, LDL = low-density lipoprotein, OR = odds ratios, P-WMH = periventricular white matter hyperintensity, SAT = subcutaneous adipose tissue.

**Table 5:** Logistic regression analyses of the association between different body composition and D-WMH.

	D-WMH			
	*P* value	OR	95% CI for OR	
			Lower	Upper
Age	.020	1.04	1.006	1.073
Male gender (%)	.436	0.79	0.441	1.423
Hypertension (%)	.212	1.41	0.821	2.427
History of CAD (%)	.346	1.39	0.700	2.770
Bone density (HU)	.097	0.99	0.986	1.001
ESMA (cm^2^)	.443	0.99	0.948	1.024

CAD = coronary artery disease, CI = confidence interval, D-WMH = deep white matter hyperintensity, ESMA = erector spinae muscle area, HU = Hounsfield attenuation units, OR = odds ratios.

## 4. Discussion

Osteosarcopenic obesity syndrome has recently been identified as a condition encompassing osteopenia/osteoporosis, sarcopenia and obesity. An important point to note is some chronic condition-related, infiltration of fat into bone marrow and muscle, probably replacing the bone and muscle cells and impairing the function of each tissue.^[9]^ It is apparent that all 3 tissues are closely interrelated and that osteopenia/osteoporosis, sarcopenia and increased adiposity need to be evaluated^[18]^ not only by BMI on obesity. Considering few study has investigated the relationship between such 3 tissues with WMH concomitantly, we explored the association between different body composition and WMH in patients with AIS in this study. Our results demonstrated that lower bone density at admission was independently associated with the moderate-severe overall WMH and P-WMH in patients with AIS which was similar to the study of Kim et al among patients with stroke.^[19]^

Age has been reported as a main risk factors for small vessel diseases.^[20]^ We found that age increased the risk of severe WMH in the univariate analyses, but after the multivariable analysis, it has only achieved statistical significance in D-WMH. As aging uniquely influences many physiological functions, the most observable are those regarding body composition changes, including loss of bone, loss of muscle mass and increased body fat.^[9]^ This might be explained that the other factors such as the loss of bone density may be an indicator of functional aging as opposed to chronological age.^[21]^

In previous studies, WMH are the most studied MRI markers of vascular brain injury and have been shown to substantially increase the risk of stroke in the general population.^[22]^ The population-based studies showed that the increased WMH volume especially P-WMH was associated with the increased risk of ischemic stroke and recurrent stroke.^[22,23]^ In our study, the history of stoke were independently associated with severe WMH and P-WMH.

Since the microvascular structures of both the kidney and the brain are similar, it has been suggested that markers for chronic kidney disease could be predictors of cerebral vascular diseases including WMH.^[24,25]^ In our study, we found that creatinine was independently associated with increased WMH as previous study.^[24,25]^

Previously, it was demonstrated that SAT which may have a protective role through the reduction of atherosclerosis was negatively associated with WMH in a neurologically healthy population.^[7]^ However, this result was not found in our AIS patients which might because of the different measurement position and distribution of SAT.

In this study, WMH was divided into P-WMH and D-WMH. A limited number of studies have investigated the differences between P-WMH and D-WMH and the underlying mechanism has not yet been fully elucidated. Our results suggest the involvement of bone density in the development of WMH and P-WMH, but not D-WMH, and the detailed mechanisms require further investigation. Previous pathology studies have shown that P-WMH is more likely to be associated with inflammation and chronic hypoperfusion, whereas D-WMH is related to ischemic damage.^[26]^ These differences may provide possible explanations for the relationship between different body composition and WMH. The proportion of moderate-severe WMH is higher than described in the literature,^[27,28]^ this could be attributed to the population studied influencing the results.

There were some limitations to this study. First, this was a cross-sectional study, so we could not establish a causal relationship between body composition such as bone density and WMH. Second, participants in our study were recruited from a single center, and this could have led to patient selection bias. Third, it is possible that other conditions/medications or residual confoundings such as diet, vitamin D or calcium intake, drugs or physical activity that could explain the observed association.^[29–31]^ Finally, we used a less precise visual rating scale to assess the degree of WMH. Quantification of WMH is needed to further investigate the relationship between body composition and WMH volume.

## 5. Conclusion

In summary, despite the above limitations, our study demonstrated a negative association between bone density and the severity of overall WMH and P-WMH WMH in AIS patients.

## Acknowledgments

The authors gratefully acknowledge the staff of the department of neurology of the Hunan University of Medicine General Hospital for their excellent support.

## Author contributions

**Conceptualization:** Xianbi Tang, Jiangli Fan, Chuzheng Pan.

**Data curation:** Fang Yu, Li Liu.

**Formal analysis:** Li Liu.

**Investigation:** Bin Wu, Dong Huang, Li Liu, Kaiquan Jing.

**Resources:** Li Liu, Xianbi Tang, Jiangli Fan.

**Supervision:** Bin Wu, Ziwei Yi.

**Validation:** Jiangli Fan.

**Writing – original draft:** Bin Wu, Dong Huang, Chuzheng Pan.

**Writing – review & editing:** Dong Huang, Ziwei Yi, Fang Yu, Chuzheng Pan.

## Supplementary Material




