# Supplementary material for: Correlation between body composition and white matter hyperintensity in patients with acute ischemic stroke
Source: Medicine (Baltimore). 2023 Dec 15;102(50):e36497. doi: 10.1097/MD.0000000000036497 (PMC10727575; doi:10.1097/MD.0000000000036497)
Supplement: Supplementary file 1 [file medi-102-e36497-s001.doc]

[Supplementary Table 1](../../../../D:%5C%E6%96%B0%E7%94%9F%E6%B4%BB%EF%BC%8C%E6%A1%8C%E9%9D%A2%5C%E7%A7%91%E7%A0%94%5C%E7%99%BD%E8%B4%A8%E9%AB%98%E4%BF%A1%E5%8F%B7%E4%B8%8E%E8%BA%AB%E4%BD%93%E7%BB%93%E6%9E%84%5C%E6%95%B0%E6%8D%AE%E9%9B%86%5CFighting%5CTable%20new%5CMedicine%5C%E8%BF%94%E4%BF%AE1%20Fighting%5C%E6%96%AD%E5%BC%80%E5%90%8E%E5%BC%95%E6%96%87.docx#S1) Baseline characteristics of all patients according to the degree of P-WMH

|  | None-mild WMH  (n=100) | Moderate-severe WMH  (n=252) | *p* |
| --- | --- | --- | --- |
| Age (years), mean±SD | 59.6 ± 11.9 | 67.7 ± 9.7 | 0.000 |
| Male gender (%) | 77 (77.0%) | 179 (71.0%) | 0.257 |
| BMI, kg/m^2^ | 24.0 ± 2.9 | 24.1 ± 3.1 | 0.709 |
| Hypertension (%) | 50 (50.0%) | 194 (77.0%) | 0.000 |
| Hyperlipidemia (%) | 45 (45.0%) | 88 (34.9%) | 0.079 |
| Diabetes mellitus (%) | 25 (25.0%) | 95 (37.7%) | 0.023 |
| History of CAD (%) | 7 (7.0%) | 37 (14.7%) | 0.049 |
| History of stroke (%) | 8 (8.0%) | 58 (23.0%) | 0.001 |
| AF (%) | 9 (9.0%) | 23 (9.1%) | 0.970 |
| Smoking (%) | 27 (27.0%) | 81 (32.1%) | 0.345 |
| Moderate or heavy drinking (%) | 12(12.0%) | 36 (14.3%) | 0.573 |
| Hypotensive drugs | 32 (32.0%) | 142 (56.3%) | 0.000 |
| Hypoglycemic drugs | 19 (19.0%) | 83 (32.9%) | 0.009 |
| NIHSS | 3.2 ± 3.4 | 3.4 ± 3.9 | 0.651 |
| Total cholesterol (mmol/L) | 5.1 ± 5.2 | 4.2 ± 1.0 | 0.009 |
| LDL cholesterol (mmol/L) | 2.7 ± 0.8 | 2.4 ± 0.9 | 0.005 |
| HDL cholesterol (mmol/L) | 1.1 ± 0.3 | 1.1 ± 0.3 | 0.878 |
| Triglyceride (mmol/L) | 1.9 ± 1.7 | 1.8 ± 1.4 | 0.548 |
| BUN (mmol/L) | 5.9 ± 2.0 | 6.3 ± 2.7 | 0.206 |
| Uric acid (umol/L) | 325.3 ± 95.2 | 336.2 ± 102.5 | 0.358 |
| Creatinine (umol/L) | 77.0 ± 21.2 | 88.9 ± 67.0 | 0.083 |
| Fasting blood-glucose (mmol/L) | 6.6 ± 3.3 | 7.1 ± 3.4 | 0.197 |
| HbA1c (%) | 7.0 ± 6.0 | 6.9 ± 2.0 | 0.757 |
| Homocysteine (umol/L) | 14.4 ± 7.7 | 15.9 ± 8.8 | 0.147 |
| Bone density (HU) | 157.0 ± 41.5 | 123.7 ± 37.3 | 0.000 |
| ESMA (cm^2^) | 34.2 ± 10.1 | 28.4 ± 7.6 | 0.000 |
| SAT (cm^2^) | 97.3 ± 45.0 | 107.6 ± 49.3 | 0.069 |
| SAT/ESMA | 3.2 ± 2.0 | 4.2 ± 2.4 | 0.000 |

BMI: body mass index; CAD: coronary artery disease; AF: atrial fibrillation; LDL: low-density lipoprotein;

HDL: high-density lipoprotein; BUN: blood urea nitrogen; SAT: subcutaneous adipose tissue;

ESMA: erector spinae muscle area.
