# Supplementary material for: Correlation between body composition and white matter hyperintensity in patients with acute ischemic stroke
Source: Medicine (Baltimore). 2023 Dec 15;102(50):e36497. doi: 10.1097/MD.0000000000036497 (PMC10727575; doi:10.1097/MD.0000000000036497)
Supplement: Supplementary file 2 [file medi-102-e36497-s002.doc]

[Supplementary Table](../../../../D:%5C%E6%96%B0%E7%94%9F%E6%B4%BB%EF%BC%8C%E6%A1%8C%E9%9D%A2%5C%E7%A7%91%E7%A0%94%5C%E7%99%BD%E8%B4%A8%E9%AB%98%E4%BF%A1%E5%8F%B7%E4%B8%8E%E8%BA%AB%E4%BD%93%E7%BB%93%E6%9E%84%5C%E6%95%B0%E6%8D%AE%E9%9B%86%5CFighting%5CTable%20new%5CMedicine%5C%E8%BF%94%E4%BF%AE1%20Fighting%5C%E6%96%AD%E5%BC%80%E5%90%8E%E5%BC%95%E6%96%87.docx#S1) 2 Baseline characteristics of all patients according to the degree of D-WMH

|  | None-mild WMH  (n=242) | Moderate-severe WMH  (n=110) | *p* |
| --- | --- | --- | --- |
| Age (years), mean±SD | 63.3 ± 11.1 | 70.0 ± 9.2 | 0.000 |
| Male gender (%) | 184 (76.0%) | 72 (65.5%) | 0.039 |
| BMI, kg/m^2^ | 24.2 ± 3.0 | 23.8 ± 3.1 | 0.251 |
| Hypertension (%) | 161 (66.5%) | 83 (75.5%) | 0.092 |
| Hyperlipidemia (%) | 97 (40.1%) | 36 (32.7%) | 0.187 |
| Diabetes mellitus (%) | 83 (34.3%) | 37 (33.6%) | 0.903 |
| History of CAD (%) | 25 (10.3%) | 19 (17.3%) | 0.068 |
| History of stroke (%) | 41 (16.9%) | 25 (22.7%) | 0.197 |
| AF (%) | 23 (9.5%) | 9 (8.2%) | 0.689 |
| Smoking (%) | 72 (29.8%) | 36 (32.7%) | 0.575 |
| Moderate or heavy drinking (%) | 32 (13.2%) | 16 (14.5%) | 0.738 |
| Hypotensive drugs | 115 (47.5%) | 59 (53.6%) | 0.287 |
| Hypoglycemic drugs | 71 (29.3%) | 31 (28.2%) | 0.824 |
| NIHSS | 3.4 ± 3.8 | 3.4 ± 3.8 | 0.933 |
| Total cholesterol (mmol/L) | 4.6 ± 3.5 | 4.2 ± 1.0 | 0.209 |
| LDL cholesterol (mmol/L) | 2.5 ± 0.8 | 2.4 ± 1.0 | 0.188 |
| HDL cholesterol (mmol/L) | 1.0 ± 0.3 | 1.1 ± 0.3 | 0.167 |
| Triglyceride (mmol/L) | 1.8 ± 1.5 | 1.6 ± 1.2 | 0.236 |
| BUN (mmol/L) | 6.1 ± 2.5 | 6.2 ± 2.6 | 0.843 |
| Uric acid (umol/L) | 332.7 ± 100.3 | 334.1 ± 101.3 | 0.906 |
| Creatinine (umol/L) | 84.4 ± 62.1 | 88.0 ± 48.0 | 0.586 |
| Fasting blood-glucose (mmol/L) | 7.1 ± 3.4 | 6.7 ± 3.3 | 0.245 |
| HbA1c (%) | 7.1 ± 4.2 | 6.7 ± 2.0 | 0.374 |
| Homocysteine (umol/L) | 15.3 ± 8.3 | 15.8 ± 8.9 | 0.651 |
| Bone density (HU) | 140.5 ± 39.7 | 117.0 ± 40.2 | 0.000 |
| ESMA (cm^2^) | 31.3 ± 8.9 | 27.2 ± 7.9 | 0.000 |
| Sat (cm^2^) | 105.8 ± 47.7 | 102.3 ± 49.7 | 0.537 |
| SAT/ESM | 3.8 ± 2.3 | 4.2 ± 2.5 | 0.115 |

BMI: body mass index; CAD: coronary artery disease; AF: atrial fibrillation; LDL: low-density lipoprotein;

HDL: high-density lipoprotein; BUN: blood urea nitrogen; SAT: subcutaneous adipose tissue;

ESMA: erector spinae muscle area.
